# Supplementary material for: Methylxanthines Inhibit Primary Amine Oxidase and Monoamine Oxidase Activities of Human Adipose Tissue
Source: Medicines (Basel). 2020 Apr 2;7(4):18. doi: 10.3390/medicines7040018 (PMC7235778; doi:10.3390/medicines7040018)
Supplement: Supplementary file 1 [file medicines-07-00018-s001.pdf]

Supplementary Materials: Methylxanthines Inhibit Primary Amine Oxidase and Monoamine Oxidase Activities of Human Adipose Tissue

Wiem Haj Ahmed, Cécile Peiro, Jessica Fontaine, Barry J. Ryan, Gemma K. Kinsella, Jeff O'Sullivan, Jean-Louis Grolleau, Gary T.M. Henehan and Christian Carpéné

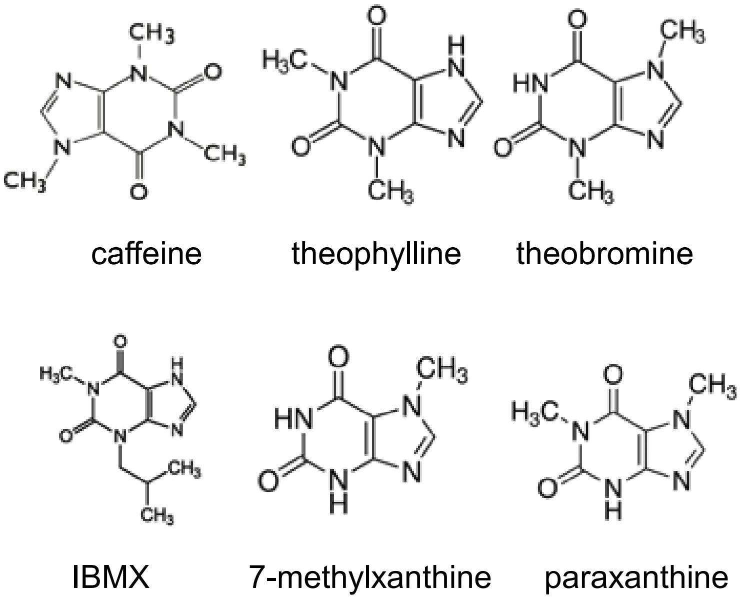

Figure S1. Chemical structures of the methylxanthines used in this study. Only 3-isobutyl-1-methylxanthine (IBMX) is not a naturally occurring N-methylated derivative of xanthine.

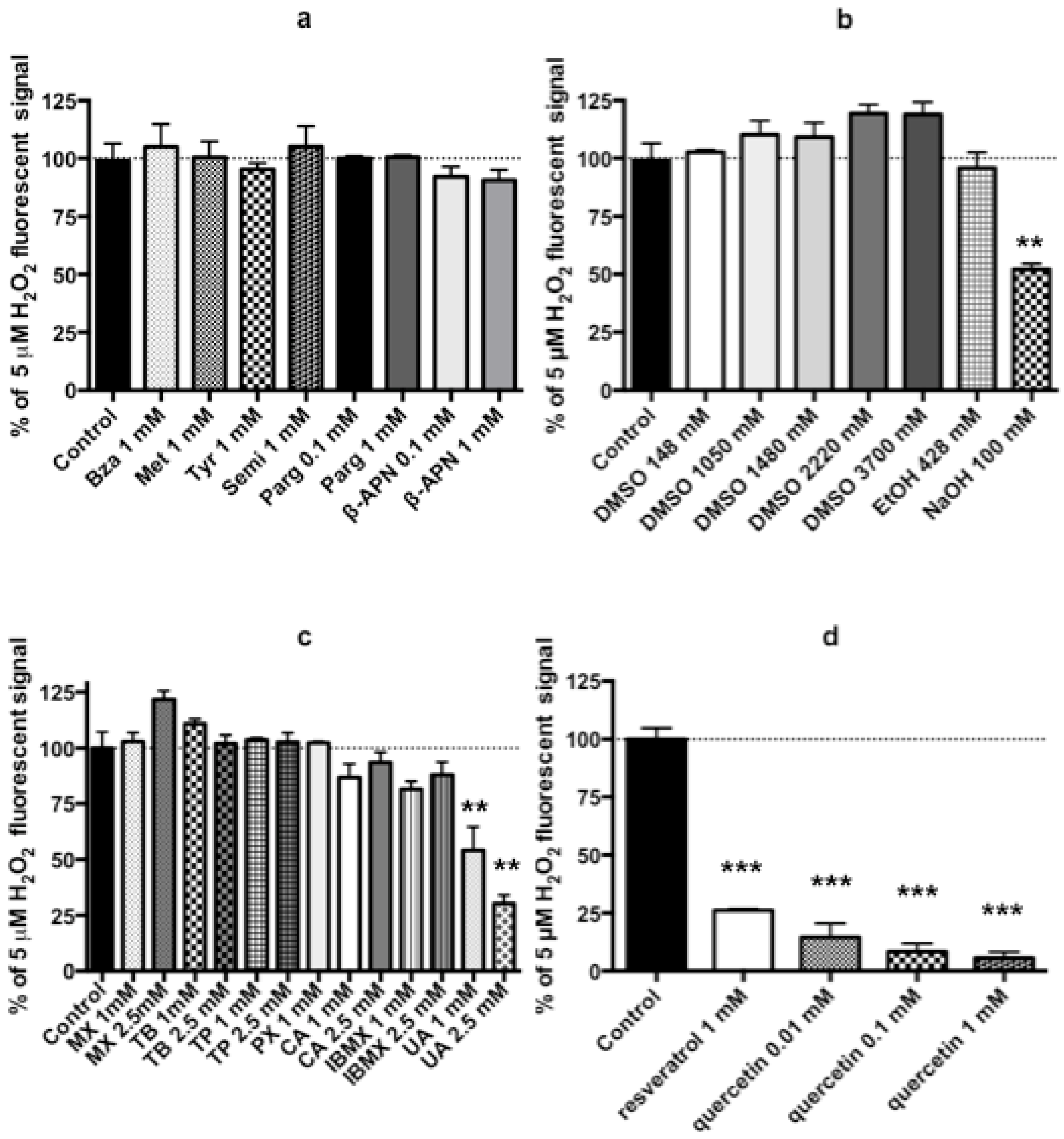

Figure S2. Interferences between Amplex Red-based fluorescent detection of hydrogen peroxide and amine oxidase substrates or inhibitors of reference (**a**); vehicles (**b**); methylxanthines (**c**); and polyphenols (**d**).The fluorescence signal of 5 μM H_2O_2 was arbitrarily set at 100% in control conditions (dotted line) and was measured in the presence of the indicated final concentrations of the following compounds: a) Bza: benzylamine; Met: methylamine; Tyr: tyramine; Semi: semicarbazide; Parg: pargyline; β-APN: β-aminopropionitrile; b) DMSO: dimethyl sulfoxide; EtOH: ethanol; c) MX: 7-methylxanthine; TB: theobromine; TP: theophylline; PX: paraxanthine; CA: caffeine; IBMX: 3-isobutyl-1-methylxanthine; UA: uric acid; d) resveratrol and quercetin. Each column is mean ± SEM of 3-8 determinations. Significantly different from control (black column) at: ** $p < 0.01$; *** $p < 0.001$.

(a)

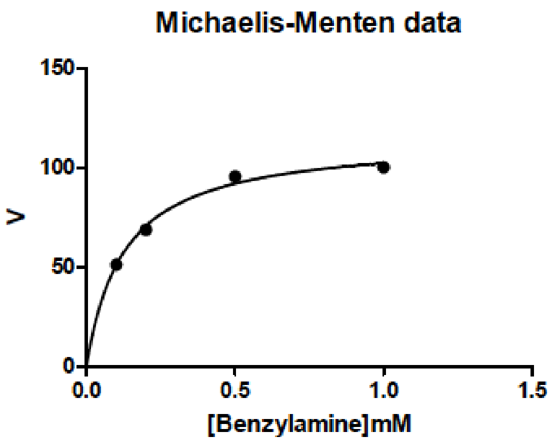
Michaelis-Menten data
150
100
50
0
V
0.0
0.5
1.0
1.5
[Benzylamine]mM

(b)

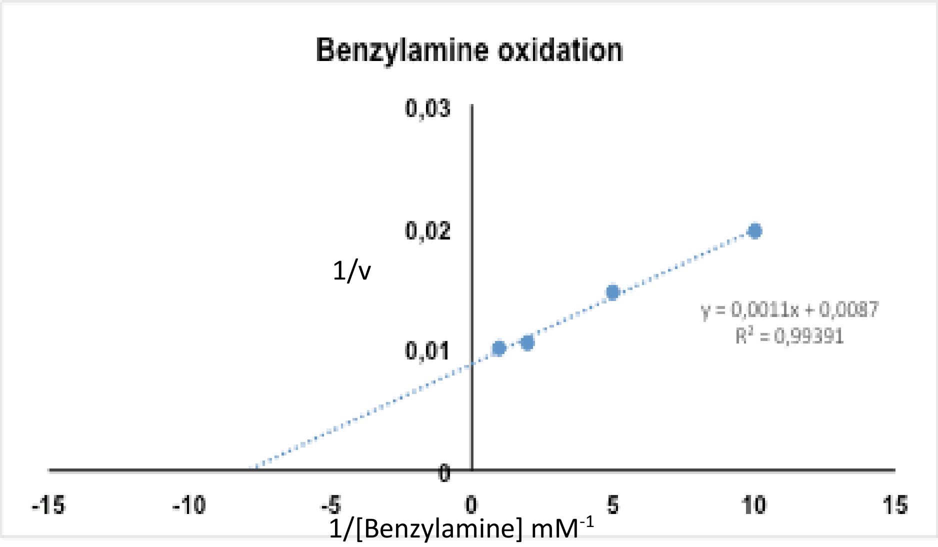
Benzylamine oxidation
0,03
0,02
0,01
0
1/v
y = 0,0011x + 0,0087
R² = 0,99391
-15
-10
-5
0
5
10
15
1/[Benzylamine] mM-1

(C)

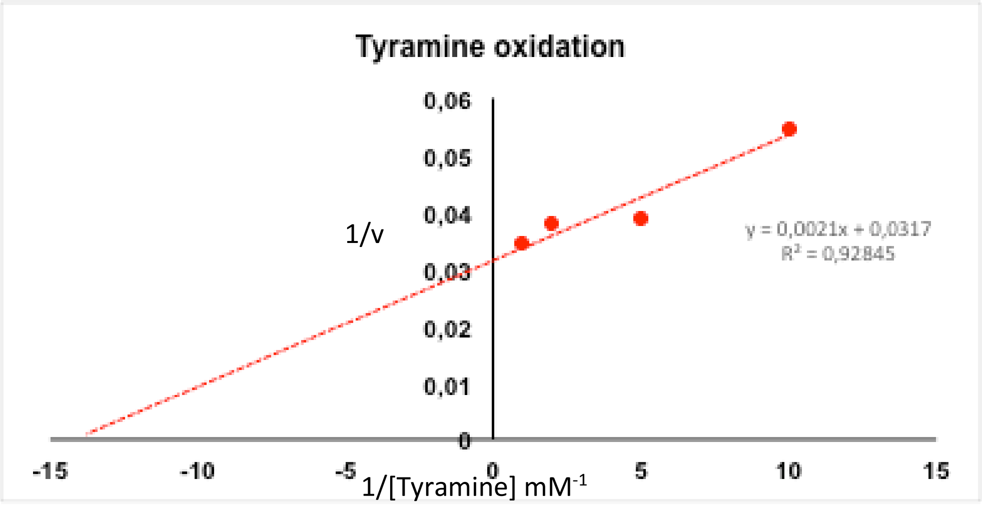

Figure S3. Michaelis-Menten data of benzylamine and tyramine oxidation by human adipose tissue homogenates. (**a**). The velocity of benzylamine oxidation (v) was determined for 30 min at final concentrations of: 0.1, 0.2, 0.5 and 1mM and plotted versus benzylamine concentration. Each point is the mean of 8 to 23 determinations. Lineweaver Burk plots with benzylamine (**b**, blue) or tyramine (**c**, red) are shown for illustrating the respective Km estimations. The Km for benzylamine was 126 µM and for tyramine it was 66 µM. Kinetic constants were estimated by nonlinear regression with the aid of Graph Pad Prism.
